# Does the age of medical emergency technicians influence the quality of chest compressions

**DOI:** 10.1186/1757-7241-22-S2-A9

**Published:** 2015-07-16

**Authors:** Svend Vittinghus, Jakob E Thomsen, Martin Harpsø, Bo Løfgren

**Affiliations:** 1 Falck Danmark A/S, Copenhagen, Demark; 2 Research Center for Emergency Medicine, Aarhus University Hospital, Aarhus, Denmark; 3 Department of Internal Medicine, Regional Hospital of Randers, Randers, Denmark; 4 Institute of Clinical Medicine, Aarhus University, Aarhus, Denmark

## Background

International resuscitation guidelines emphasize performance of high quality chest compressions. In out-of-hospital cardiac arrest Emergency Medical Technicians (EMT) play an important role in delivering chest compressions. Delivery of chest compressions is physically demanding and the quality of chest compressions is related to the fitness of the rescuer. Physical fitness may decrease with age. It is currently unknown whether age of professional rescuers influences the quality of chest compressions. The aim of this study was to investigate if the age of EMTs influences quality of chest compressions.

## Method

EMTs trained in the 2010 resuscitation guidelines delivered uninterrupted chest compressions for 6 min on a mannequin. Data were collected from the mannequin on a laptop and from video recordings. EMTs were divided into three groups: <36 (young), 36-50 (middle age) and >50 (oldest) years. Primary outcome measure: chest compression rate and depth measured for the first 2 (0-2) min of chest compressions. Secondary outcome measure: chest compression rate and depth measured for the last 2 (4-6) min.

## Results

In total, 86 EMTs were included (young, n = 26; middle age, n = 37; oldest, n = 23). During the first 2 (0-2) min, chest compression depth tended (ANOVA, p = 0.08) to be lower among the oldest (mean ± SD: oldest: 43 ± 12 mm; middle age: 49 ± 9 mm; young: 46 ± 10 mm). Chest compression rate was significantly higher among the oldest (118 ± 15 min^-1^) when compared to the middle age (106 ± 14 min^-1^, p < 0.01) but not significant different from the young (112 ± 13 min^-1^). In the final 2 (4-6) min, chest compression depth was lower among the oldest (41 ± 13 mm) compared to middle age (48 ± 10 mm, p < 0.05) but not the young (43 ± 9 mm). In contrast, chest compression rate was significantly higher among the oldest (118 ± 16 min^-1^) when compared to the middle age (104 ± 17 min^-1^, p < 0.05) but not significantly different from the young (114 ± 17 min^-1^).

## Conclusion

Age influence chest compression quality parameters among EMTs. There is a tendency to a clinical significant reduction in chest compression depth among the oldest EMT. Mean chest compression depth was too low among all groups of EMTs underlining the importance of CPR training and regular retraining.

